# Epstein-Barr Functional Mimicry: Pathogenicity of Oncogenic Latent Membrane Protein-1 in Systemic Lupus Erythematosus and Autoimmunity

**DOI:** 10.3389/fimmu.2020.606936

**Published:** 2021-02-03

**Authors:** Melissa E. Munroe, Jourdan R. Anderson, Timothy F. Gross, Laura L. Stunz, Gail A. Bishop, Judith A. James

**Affiliations:** ^1^ Arthritis and Clinical Immunology Program, Oklahoma Medical Research Foundation, Oklahoma City, OK, United States; ^2^ Department of Microbiology & Immunology, The University of Iowa, Iowa City, IA, United States; ^3^ Department of Internal Medicine, The University of Iowa, Iowa City, IA, United States; ^4^ Holden Comprehensive Cancer Center, The University of Iowa, Iowa City, IA, United States; ^5^ Iowa City VA Medical Center, Iowa City, IA, United States; ^6^ Department of Medicine and Pathology, Oklahoma University Health Sciences Center, Oklahoma City, OK, United States

**Keywords:** autoimmunity, systemic lupus erythematosus, Epstein-Barr virus, molecular mimicry, functional mimicry, EBNA-1, LMP1, mouse

## Abstract

Systemic lupus erythematosus (SLE) and other autoimmune diseases are propelled by immune dysregulation and pathogenic, disease-specific autoantibodies. Autoimmunity against the lupus autoantigen Sm is associated with cross-reactivity to Epstein-Barr virus (EBV) nuclear antigen 1 (EBNA-1). Additionally, EBV latent membrane protein-1 (LMP1), initially noted for its oncogenic activity, is an aberrantly active functional mimic of the B cell co-stimulatory molecule CD40. Mice expressing a transgene (Tg) for the mCD40-LMP1 hybrid molecule (containing the cytoplasmic tail of LMP1) have mild autoantibody production and other features of immune dysregulation by 2–3 months of age, but no overt autoimmune disease. This study evaluates whether exposure to the EBV molecular mimic, EBNA-1, stimulates antigen-specific and concurrently-reactive humoral and cellular immunity, as well as lupus-like features. After immunization with EBNA-1, mCD40-LMP1 Tg mice exhibited enhanced, antigen-specific, cellular and humoral responses compared to immunized WT congenic mice. EBNA-1 specific proliferative and inflammatory cytokine responses, including IL-17 and IFN-γ, were significantly increased (*p<0.0001*) in mCD40-LMP1 Tg mice, as well as antibody responses to amino- and carboxy-domains of EBNA-1. Of particular interest was the ability of mCD40-LMP1 to drive EBNA-1 associated molecular mimicry with the lupus-associated autoantigen, Sm. EBNA-1 immunized mCD40-LMP1 Tg mice exhibited enhanced proliferative and cytokine cellular responses (p<0.0001) to the EBNA-1 homologous epitope PPPGRRP and the Sm B/B’ cross-reactive sequence PPPGMRPP. When immunized with the SLE autoantigen Sm, mCD40-LMP1 Tg mice again exhibited enhanced cellular and humoral immune responses to both Sm and EBNA-1. Cellular immune dysregulation with EBNA-1 immunization in mCD40-LMP1 Tg mice was accompanied by enhanced splenomegaly, increased serum blood urea nitrogen (BUN) and creatinine levels, and elevated anti-dsDNA and antinuclear antibody (ANA) levels (*p<0.0001* compared to mCD40 WT mice). However, no evidence of immune-complex glomerulonephritis pathology was noted, suggesting that a combination of EBV and genetic factors may be required to drive lupus-associated renal disease. These data support that the expression of LMP1 in the context of EBNA-1 may interact to increase immune dysregulation that leads to pathogenic, autoantigen-specific lupus inflammation.

## Introduction

Systemic lupus erythematosus (SLE) is a chronic autoimmune disease driven by dysregulated cellular and humoral immunity (1–4). Increased immune dysregulation is associated with increased clinical disease activity and flare (5–7) and places patients at risk of permanent end-organ damage, SLE-associated morbidity, and early mortality (8). Such immune dysregulation begins years before clinical disease onset and amplifies through a feed-forward aggregation of altered innate and adaptive immune pathways as patients progress to SLE classification. Concurrent with or following these changes in innate and adaptive immune pathways, pathogenic SLE- associated autoantibody specificities accumulate (9). These autoreactive responses commonly target nuclear antigens such as Ro/SSA, La/SSB, Sm, RNP, and dsDNA (3, 4), the latter two specificities associated with lupus nephritis (10). Despite improved disease management and treatment approaches to suppress and circumvent dysregulated immunity, patients with SLE exhibit persistent and waxing/waning dysregulation of innate and adaptive immune pathways.

Numerous studies over the past two decades have elucidated genetic and genomic contributions to SLE risk and heritability. Despite a twin concordance rate of up to 25% (11) and identification of over 100 lupus associated genetic variants (12, 13), genetics alone explain no more than 50% of SLE risk (14, 15). This supports roles for environmental factors as contributors to SLE etiology (16, 17). Infections, such as Epstein-Barr virus (EBV), are associated with both pediatric (18, 19) and adult (17, 20, 21) SLE. EBV, a member of the herpes virus family, is tropic for B-lymphocytes and promotes cellular dysregulation, including lymphoproliferation (22–24), malignancy (25–27), and autoimmunity (28–30). Compared to unaffected individuals, SLE patients have higher EBV viral loads (31, 32), are more likely to exhibit infection in peripheral blood mononuclear cells (PBMCs) (32, 33) and exhibit aberrant expression of EBV latent genes, including EBV nuclear antigen-1 (EBNA-1) and latent membrane protein-1 (LMP1). These differences in SLE patients may be attributed to immune dysregulation that drives latent protein expression as well as an inability to control viral reactivation (17, 19, 24, 33–37). EBV reactivation is increased in SLE patients, evidenced by increased antibodies to EBNA-1 in conjunction with IgG antibodies against EBV early antigen (EA) and viral capsid antigen (VCA) (17, 38). This viral reactivation is associated with transition to classified SLE (17) as well as clinical disease activity and flare (18, 31).

Both pediatric and adult SLE patients exhibit altered humoral immunity to EBNA-1 (9, 19, 39, 40). EBNA-1 is a structural, molecular mimic with known SLE autoantigens. By eliciting antibodies that structurally cross-react with autoantigens, we and others have shown that EBNA-1 contributes to autoimmunity against Ro/SSA and spliceosomal proteins Sm B, Sm D1, and RNP A (9, 19, 40–42); additional studies by the Spatz laboratory have further found cross-reactivity between EBNA-1 and the SLE-associated autoantigen dsDNA (42–44). Although structural molecular mimicry may be due to random chance, EBNA-1 utilizes and binds to the same nuclear spliceosomal machinery as host cells (45), including Sm and RNP proteins (46), to maintain lytic and latent EBV infection (47). Therefore, functional and structural overlap may drive molecular mimicry between EBNA-1 and SLE-associated autoantigens. Over time with continued cross-reactivity, broken immune tolerance creates a positive feedback loop where autoantibodies mediate cellular damage that releases additional autoantigens, leading to continued immune reactivity, epitope spreading (35, 48), and the accumulation of autoantibody specificities (3, 4, 49) that themselves cross react with EBNA-1 (35). Anti-EBNA-1 antibodies alone may not be enough to break tolerance and drive autoimmunity, as over 90% of individuals have been exposed to EBV and most never develop autoimmunity (50, 51).

**Figure 1:**
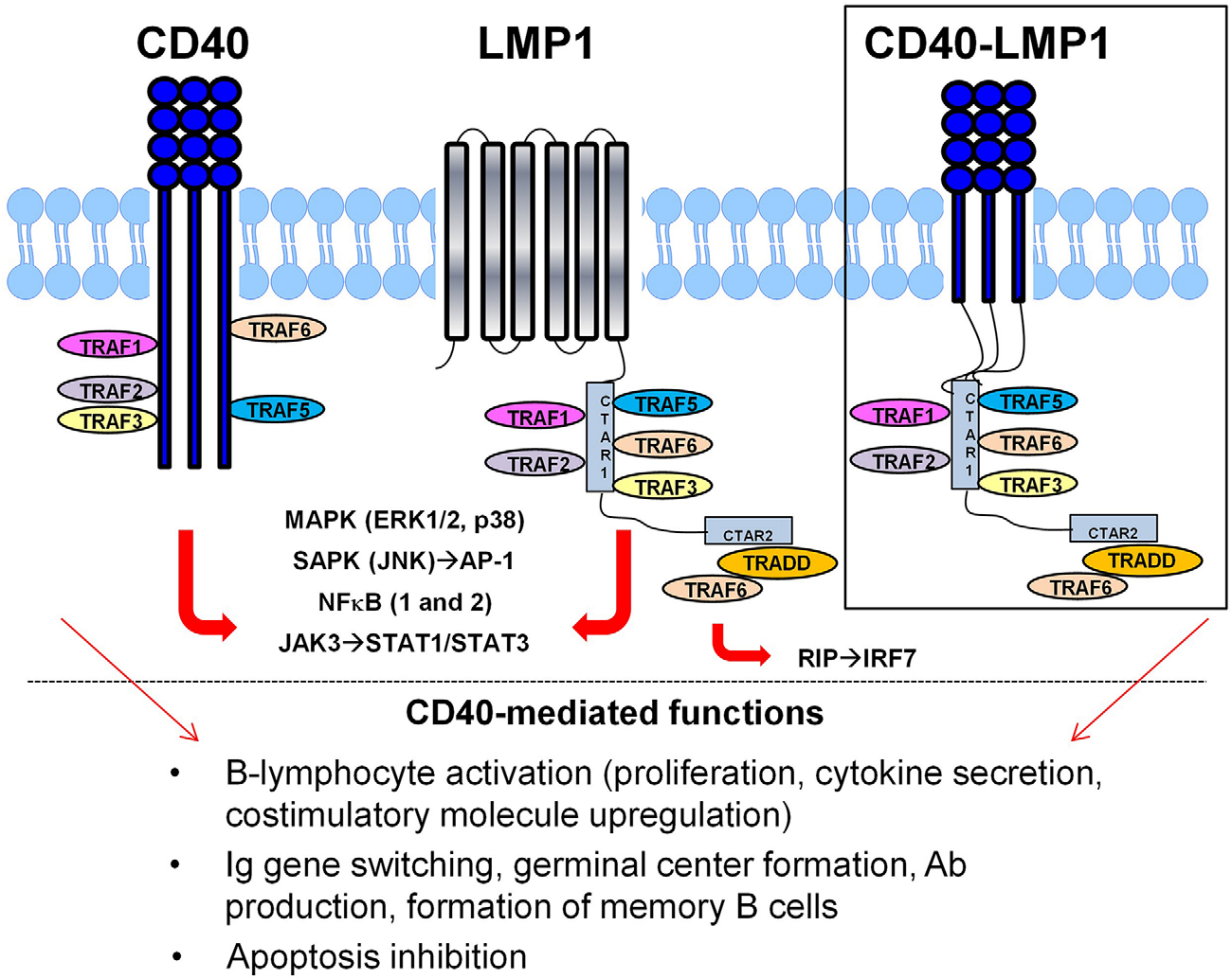
Latent Membrane Protein 1 (LMP1) acts as a viral mimic of the costimulatory molecule CD40. Similar to CD40, LMP1 binds TRAFs through its cytoplasmic domain to mediate proximal signaling/transcriptional regulation and downstream function, including B-lymphocyte activation, antibody production and isotype switching, and apoptosis inhibition. However, LMP1 does this in a dysregulated manner, partially through its ligand-independent, self-aggregating six-transmembrane domains. Replacing the extracellular/transmembrane domain of LMP1 with CD40 demonstrates that the cytoplasmic tail of LMP1 is necessary and sufficient for its enhanced and dysregulated functional mimicry of CD40 (inset).

Cellular immune dysregulation may facilitate the initial break in tolerance in SLE, as SLE-associated autoantibody specificities associated with EBNA-1 molecular mimicry are detected after evidence of cellular immune dysregulation in pre-clinical SLE (3, 4). Interestingly, EBV encodes proteins that disrupt cellular immune regulation, including LMP1, a functional mimic of CD40. As a costimulatory molecule expressed on antigen-presenting cells, such as B-lymphocytes, dendritic cells, and macrophages, CD40 is vital for B-lymphocyte activation and function and bridges innate and adaptive immunity. Interacting with CD154 on T-lymphocytes (52), CD40 itself triggers B-lymphocyte activation, proliferation, cytokine secretion, and antibody production (52), acts as a co-stimulatory molecule for the B cell receptor (BCR) (53, 54), and amplifies innate signals driven by toll like receptors (TLRs) (55), including TLR7 (56, 57), implicated in SLE pathogenesis (58–60). EBV-encoded LMP1 has been studied *in vitro* (61–66) and *in vivo* (62, 67–71) and is a functional mimic of CD40, although it does so in an enhanced and dysregulated manner (**Figure 1**). Unlike CD40, which requires interaction and trimerization with CD154, the six transmembrane domains of LMP1 are able to self-aggregate in a ligand-independent and uncontrolled manner to drive downstream proximal signaling and subsequent distal functional activities that overlap with CD40, including B-lymphocyte activation, germinal center formation, as well as antibody and cytokine production (71). This ability of LMP1 to spontaneously self-aggregate, without the need for CD154 expressed on T-lymphocytes, may allow for its ability to evade the immune system and contribute to the natural selection of EBV to latently persist within B-lymphocytes (72, 73).

Like CD40, the cytoplasmic domain of LMP1 does not have enzymatic activity, but instead utilizes TNF-receptor associated factors (TRAFs) to facilitate its signaling and biologic activities. Replacing the LMP1 extracellular/transmembrane domains with those of CD40 (**Figure 1** **, inset**) demonstrated that the cytoplasmic tail of LMP1 is necessary and sufficient to mimic CD40 activity and do so in a dysregulated manner (71, 74). LMP1 interacts with TRAFs *via* two carboxy-terminus activating regions (CTAR), CTAR1 and CTAR2 (66, 68). CTAR1, similar to CD40, contains the TRAF binding motif, PXQXT, to bind TRAF1, TRAF2, TRAF3, TRAF5, and TRAF6 (63, 68). Yet there are key differences in the way LMP1 utilizes TRAFs compared to CD40. CD40 drives B-lymphocyte activation primarily through TRAFs 2 and 6 (75, 76), as well as TRAF1 (77), with TRAF3 acting as an *inhibitor* (65, 78). In contrast, LMP1 utilizes TRAF3 (63–65) in an *activating* manner alongside TRAF5 (62), as well as TRAFs 1 and 2 (79, 80). Furthermore, CD40-mediated signaling results in ubiquitination and degradation of TRAFs 2 and 3 to downregulate its signal; this does *not* occur in LMP1 signaling (63, 74). In addition, LMP1 indirectly binds TRAF6 *via* TRADD in its CTAR2 domain (81, 82), allowing for additional CD40 signals *via* IRAK1 (83), as well as IRF7 activation *via* RIP (84).

These dysregulated, pro-activation differences in utilization of TRAFs by LMP1 have been shown to translate into an autoimmune disease phenotype *in vivo*. The mCD40-LMP1 transgenic (Tg) mouse model expresses a hybrid molecule with the mouse (m)CD40 extracellular domains and the LMP1 cytoplasmic tail, as described above. The transgene is driven by an MHCII promoter on a C57BL/6 (B6), CD40-deficient background, so that the only CD40 present is mCD40-LMP1. Compared to congenic mCD40 Tg and B6 mice that express full-length, wild-type mCD40 (mCD40 WT mice), mCD40-LMP1 Tg mice exhibit both splenomegaly and lymphadenopathy, with expanded immature/activated B-lymphocyte populations and ectopic germinal center formation. In addition, these mice produce autoantibodies, including anti-dsDNA, and exhibit aberrant cytokine levels, including IL-6 (62, 67, 68, 71). Yet, the mCD40-LMP1 Tg mice are capable of driving T-dependent antibody responses, with normal isotype switching, affinity maturation, and germinal center formation (71).

We have previously demonstrated that in the context of type II collagen, an autoantigen that induces inflammatory arthritis in a murine model of rheumatoid arthritis (85), mCD40-LMP1 Tg mice exhibit accelerated and exacerbated inflammatory arthritis compared to their congenic WT counterparts (70). *Ex vivo*, these mice exhibit enhanced innate and adaptive cellular immunity in antigenic recall responses, particularly TNF-α, IL-6, and IL-17A, as well as enhanced TNF-α and IL-6 secretion in activated B-lymphocytes. This enhanced cellular immunity is accompanied by an increase in total and collagen-specific antibody production (70), with immune pathway specific isotype switching, suggesting that LMP1 is able to drive enhanced and dysregulated cellular and humoral adaptive immunity.

Because mCD40-LMP1 drives an autoimmunity phenotype that leads to overt pathology in the context of the autoantigen collagen (70), we hypothesized that LMP1 may enhance the onset of autoimmunity in conjunction with molecular mimicry between EBNA-1 and the SLE-associated autoantigen, Sm (86). Therefore, the current study investigates antigen-specific cellular and humoral immune responses to EBNA-1 and its cross-reactive lupus autoantigen, Sm, in the context of mCD40-LMP1-mediated adaptive immunity. Based on our previous epitope mapping studies, this includes reactivity in EBNA-1 and Sm immunized mice to the antigenic epitope, PPPGRRP (EBNA-1) and its homologous, comparable antigenic epitope sequence, PPPGMRPP (Sm) (19, 51, 86, 87). Further, we evaluated these mice for enhanced splenomegaly, the presence of ANA and anti-dsDNA autoantibodies, and altered renal function.

## Materials and Methods

### Mice

C57BL/6 mice were purchased at 5–8 weeks of age from the National Cancer Institute (Bethesda, MD). Mice transgenic for the molecule mCD40-LMP1 (mCD40-LMP1 Tg) or full length mCD40 (mCD40 Tg), driven by the MHC Class II Eα promoter were transferred from the Bishop Lab (The University of Iowa) to the Oklahoma Medical Research Foundation (OMRF). In addition to B-lymphocytes, EBV can also infect myeloid cells (88, 89), so it is reasonable to express LMP1 on these cell types and B-lymphocytes. Tg mice were maintained on the C57BL/6 CD40-deficient background (B6.129P2-CD40tm1Kik/J from The Jackson Laboratory, Sacramento, CA) at OMRF, as previously described (71). Mice were age- and sex-matched and analyzed at 3–4 months of age. All mice were housed in specific pathogen-free barrier facilities with restricted access, all animal care and housing requirements of the National Institutes of Health Committee on Care and Use of Laboratory Animals were followed, and all procedures were approved by the OMRF Animal Care and Use Committee.

### Immunizations

**Figure 2:**
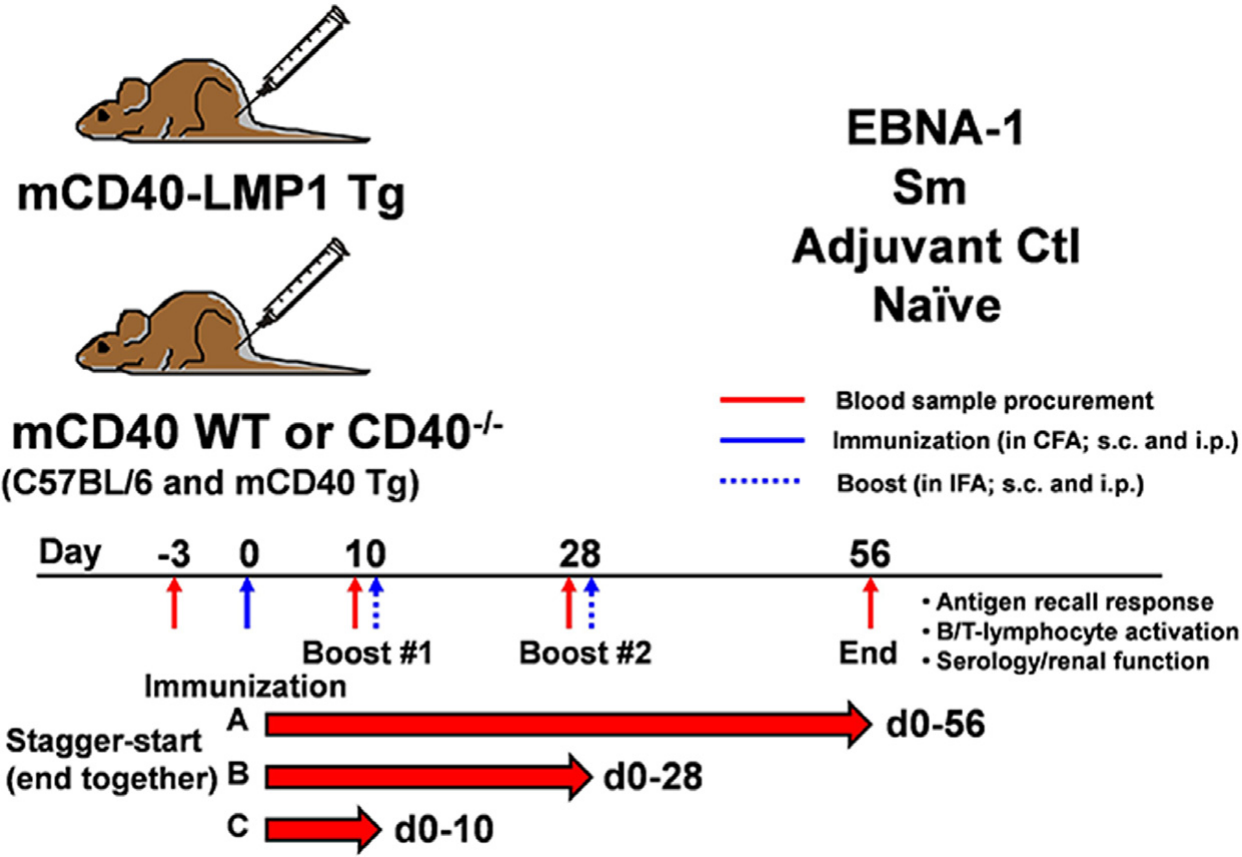
Immunization of mCD40-LMP1 Tg mice with EBNA-1, Sm, or controls to assess antigen-specific immune response and antigen cross-reactivity. As described in *Materials and Methods*, mCD40-LMP1 Tg, mCD40 WT (C57BL/6 and mCD40 Tg), and mCD40^-/-^ mice remained naïve or were immunized with either saline (adjuvant only), EBNA-1, or Sm on Day 0 in CFA, then received boost injections (in IFA) on Days 10 and Day 28. Mice were euthanized on Day 56, spleen and lymph nodes removed, and cell cultures completed. Blood samples were procured for serum prior to initial immunization (Day -3), and on Days 10, 28, and 56 after initial immunization. Separate groups of EBNA-1 immunized mice completed Days 0–10 or Days 0–28 of the protocol. Each experimental group contained 6–8 mice across two separate experiments.

Mice were immunized based on protocols described (86, 90) (**Figure 2**). Briefly, all mouse strains either remained naïve (n=6 mice/strain) or were immunized with sterile saline (adjuvant control; n= 6 mice/strain), 100 µg EBNA-1 mosaic (n = 8 mice/strain; EBNA-1 antigen with truncated glycine-alanine repeat; BiosPacific, Inc./Bio-techne, Emeryville, CA), or 100 µg Sm antigen (n= 6 mice/strain; Immunovision; Springdale, AR). Saline and immunogens were emulsified 1:1 in either Complete Freund’s Adjuvant (CFA; Sigma-Aldrich/MilliporeSigma, St. Louis, MO) for initial immunization (Day 0), or Incomplete Freund’s Adjuvant (IFA; Sigma-Aldrich/MilliporeSigma) for booster immunizations (Day 10, Day 28). Emulsified adjuvant control (saline) and immunogens were injected in equal portions intraperitoneally (50 µg/100µl) and subcutaneously in alternating flanks (50µg/100µl). Blood samples for sera were collected on Days -3, 10, 28, and 56 relative to initial immunization *via* tail vein sampling. A subset of EBNA-1 immunized mice completed 10 or 28 days (n = 6 mice/strain) of the protocol (mice groups were staggered so that all of the mice completed the experimental protocol on the same day).

### Lymph Node (LN) Cell Culture

Single cell suspensions (4 x 10^6^/ml) of axillary, mesenteric, and inguinal draining LNs from mice were cultured in RPMI 1640 with 5% heat-inactivated fetal calf serum (FCS; VWR International, Radnor, PA), 10 uM 2-mercaptoethanol (Life Technologies/Thermo Fisher Scientific, Waltham, MA), penicillin and streptomycin. Cells were cultured in medium alone or in the presence of 50 ug/ml EBNA-1 mosaic (EBNA-1 with truncated glycine-alanine rich region), Sm antigen, the EBNA-1 homologous antigenic peptide PPPGRRP, the Sm homologous antigenic peptide PPPGMRPP, or 5 ng/ml PMA + 500 ng/ml ionomycin (positive control; purchased from Sigma-Aldrich/MilliporeSigma). Bulk quantities of the peptides PPPGRRP and PPPGMRPP were constructed on polylysine backbones (MAP™, Applied Biosystems, Foster City, CA) by the University of Oklahoma Health Sciences Center Molecular Biology-Proteomics facility. Antigen specific proliferation was determined in 72 h 96-well cultures by pulsing with 1 µCi/well [3H]TdR (GE Healthcare/Amersham Biosciences) at 48 h, and cpm was determined by liquid scintillation 24 h later. Culture supernatants were collected at optimal culture times for cytokine analysis: 48 h for IL-6, TNF-α, and IL-10, and 72 h for IFN-γ and IL-17A.

### Cytokine ELISA

Cytokine concentrations in culture supernatants were determined by ELISA, using cytokine-specific coating and biotinylated detection antibodies diluted per manufacturer’s protocol (eBioscience/Invitrogen/Thermo Fisher Scientific). Streptavidin-HRP (Jackson ImmunoResearch Laboratories, Inc., West Grove, PA) binding to biotinylated detection antibodies was visualized with TMB substrate (KPL/Seracare, Milford, MA) and the reaction was stopped with 0.18 M H_2_SO_4_. Plates were read at 450 nm *via* Emax Plus Reader (Molecular Devices, San Jose, CA). Data were analyzed with SoftMax Pro software (Molecular Devices); unknowns were compared with a standard curve containing at least five to seven dilution points of the relevant recombinant cytokine (eBioscience/Invitrogen/Thermo Fisher Scientific) on each assay plate. In all cases, the coefficient of determination for the standard curve (*r* ^2^) was ≥0.98. ELISA unknowns were diluted to fall within the range of standard values.

### Anti-EBNA-1 and Anti-Sm Serology

Standard solid-phase assays were used to measure the antibody reactivity in mouse sera, as described previously (86). One µg of Sm (Immunovision, Springdale, AR) or EBNA-1 mosaic (BiosPacific) was coated per well in each of 96 polystyrene wells/plate. Mouse sera at a dilution of 1:100 (Sm) or 1:1000 (EBNA-1) were incubated in each well for 3 hrs. After incubation, plates were washed and incubated with anti-mouse alkaline phosphatase-conjugated γ-chain-specific goat IgG (Sigma-Aldrich/MilliporeSigma) at 1/10,000 dilution. Para nitrophenyl phosphate disodium (PNPP, Sigma-Aldrich/MilliporeSigma) was used as a substrate for alkaline phosphatase, and plates were read at 405 nm *via* Emax Plus reader (Molecular Devices). ELISA tests were considered positive if the optical density (OD) was at least two standard deviations above the naïve/adjuvant control mean.

### Solid-Phase Peptide Synthesis and Anti-Peptide Assays

Sequential, overlapping octapeptides from EBNA-1 and Sm BB’ were synthesized at the ends of radiation-derivatized polyethylene pins arranged in a 96-well microtiter plate format, as described previously (19, 51). All unique octapeptides (EBNA-1 aa 1–103 and 288–641; Sm BB’ aa 1-233) were synthesized, while duplicate octapeptides (especially in the glycine–alanine-rich region of EBNA-1 [aa 97–321]) were omitted. Positive control pins were synthesized from a known reactive sequence of the Sm B’ protein (PPPGMRPP) and used with previously characterized reactive (positive) and non-reactive (negative) sera as standards. Sera from mCD40-LMP1 Tg, mCD40 WT, and CD40-deficient mice were tested for binding with the EBNA-1 or Sm BB’ octapeptides by a solid-phase ELISA-based immunoassay, as previously described (19, 51, 86, 91). Briefly, individual solid-phase peptides were incubated with a 1:100 dilution of mouse sera for 2 h at room temperature. Each pin block was washed and incubated with anti-mouse IgG Fc-specific alkaline phosphatase conjugate or with anti-human IgG alkaline phosphatase conjugate for the positive controls (Jackson Immunoresearch Laboratories), overnight at 4°C. Pin blocks were washed, then incubated at 37°C with PNPP substrate until positive control wells had absorbance readings of 1.0 at 405 nm. A well-characterized human positive control serum was used to normalize the results among multiple plates. Reactivity against an octapeptide was considered positive if the absorbance was at least four standard deviations above the naïve/adjuvant control mean.

### Autoantibody Detection and Renal Function Tests

Sera were assessed for anti-nuclear antibodies (ANA; Alpha Diagnostic International, San Antonio, TX), anti-dsDNA antibodies (Alpha Diagnostic Int’l), blood urea nitrogen (BUN; Arbor Assays, Ann Arbor, MI), and serum creatinine (Arbor Assays) per manufacturers’ protocols. For ANA and anti-dsDNA assays, sera were measured in duplicate at a 1:100 dilution in a 96-well plate format, and the HRP-coupled secondary Ab was goat anti mouse IgG (H and L). Negative and positive control sera, as well as 5 point calibration curve samples, provided by the manufacturer, were run concurrently with the unknown samples. Sera were diluted 1:10 for BUN assays and 1:30 for creatinine assays, per manufacturers’ protocols. Sera were run in duplicate alongside a 5 (creatinine) or 7 (BUN) point standard curve. All assays were read at 450 nm using an Emax Plus Reader (Molecular Devices). Unknowns were compared with a calibration curve containing five dilution points on each assay plate. In all cases, the coefficient of determination for the standard curve (*r* ^2^) was ≥0.98.

### Statistical Analyses

Analyses were performed with GraphPad version 7.02 Instat software. Student’s paired t-test was used to determine significance between paired groups. One-way ANOVA with Dunnett’s multiple comparison test was used to determine significance between >2 groups. P-value ≤0.05 was considered significant.

## Results

### Assessment of LMP1 Functional Mimicry in the Context of EBNA-1

We have previously demonstrated that mCD40-LMP1 Tg mice exhibit mild autoimmunity, marked by lymphadenopathy, splenomegaly, enhanced cytokine secretion, and autoantibody production (71). We therefore asked how mCD40-LMP1 would influence antigen-specific inflammatory responses and lupus-like pathogenic features in the context of EBNA-1. Based on our previous studies assessing EBNA-1 humoral immunity in animal models (86, 90), mCD40-LMP1 Tg mice and congenic controls (B6, mCD40Tg, and B6.CD40-deficient mice) were immunized (in CFA) with EBNA-1 or its cross-reactive autoantigen, Sm, and boosted (in IFA) over a 56-day course (**Figure 2**). Additional mice completed either a 10-, or 28-day EBNA-1 immunization/booster protocol to determine cellular and humoral immune response kinetics. Sera were collected for serology and renal function testing, lymph nodes for assessment of antigen recall responses, and spleens for assessment of splenomegaly and activation capacity of T- and B-lymphocytes.

### mCD40-LMP1 Tg Mice Mount Accelerated and Enhanced Cellular Immune Response to EBNA-1 Immunization

**Figure 3:**
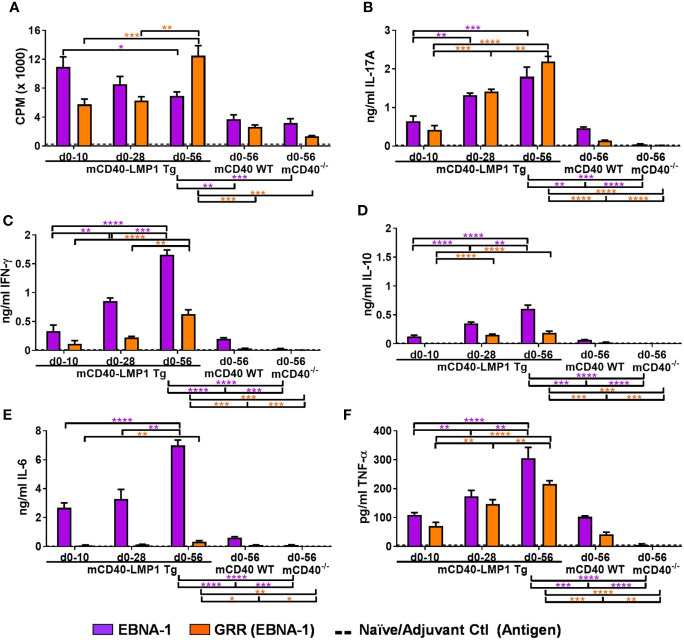
Enhanced antigen recall response to EBNA-1 and homologous region PPPGRRP in mCD40-LMP1 Tg mice. Lymph node cells (4x 10^6^/ml) were cultured in the presence of 50 µg/ml EBNA-1 (mosaic) vs. 50 µg/ml PPPGRRP (GRR; EBNA-1 antigenic epitope). Cell cultures were assessed for proliferation **(A)** and cell culture supernatant IL-17A **(B)**, IFN-γ **(C)**, IL-10 **(D)**, IL-6 **(E)**, and TNF-α **(F)**. Antigen recall response from mCD40-LMP1 Tg mice was compared to that of CD40 WT mice and CD40^-/-^ mice (**Figure 2**). Data presented as mean ± SEM. *p ≤ 0.05, **p ≤ 0.01, ***p < 0.001, ****p < 0.0001 one way ANOVA with Dunnett’s multiple comparison test. Significance between experimental groups of mice designated above bar graphs (mCD40-LMP1 mice over time) and below bar graphs (mCD40-LMP1 mice vs. CD40 WT vs. CD40^-/-^ mice); purple = EBNA-1; orange = GRR. A minimal cellular response was exhibited by naïve/adjuvant control mice (dotted line near bottom of y-axis). A minimal cellular response was exhibited by d0-10 and d0-28 mCD40 WT and CD40-deficient mice; only d0-56 data are shown. Antigenic stimulation vs. medium only and PMA/ionomycin is presented in **Supplementary Figure 1**.

To compare the cellular immune response to EBNA-1 and its antigenic epitope PPPGRRP, homologous to a comparable sequence in the lupus autoantigen Sm, draining lymph node cells from EBNA-1-immunized mCD40-LMP1 Tg, congenic WT, or CD40-deficient mice were cultured in the presence or absence of antigen (**Figure 3** and **Supplementary Figure 1**). All three strains of mice were able to mount a proliferative antigen recall response against EBNA-1 56 days after initial EBNA-1 immunization (**Figure 3A**). However, mCD40-LMP1 mice showed a significantly greater response to EBNA-1 (*p<0.01*) and PPPGRRP (*p<0.001*), even after the proliferative response in mCD40-LMP1 mice shifted over time away from an EBNA-1 antigenic response toward PPPGRRP (from 10 to 28 to 56 days post-immunization). Similarly, all strains of mice produced cytokines after PMA/ionomycin stimulation as a positive control, but antigen-specific cytokine secretion was significantly enhanced in mCD40-LMP1 mice, with some unique differences between EBNA-1 and PPPGRRP stimulation (**Figures 3B–F**). Both EBNA-1 and PPPGRRP stimulated high levels of IL-17 (**Figure 3B**) and IFN-γ (**Figure 3C**) in EBNA-1-immunized mCD40-LMP1 mice, where IL-17 and IFN-γ responses increased over time, reaching the maximum levels seen in day 56 WT mice by day 10. The IL-10 response (**Figure 3D**) was also elevated, though relatively delayed compared to IL-17 and IFN-γ. Interestingly, IL-6 was secreted much more robustly in response to EBNA-1 than to PPPGRRP in mCD40-LMP1 mice, with minimal response to either antigen in CD40 WT mice (**Figure 3E**). Conversely, TNF-α secretion increased after both EBNA-1 and PPGRRP antigenic stimulation as early as 10 days post-immunization in mCD40-LMP1 mice and by day 56 in mCD40 WT mice, but not in CD40-deficient mice (**Figure 3F**).

### Concurrently Reactive Cellular Immune Response Between EBNA-1 and Sm in EBNA-1 Immunized mCD40-LMP1 Mice

**Figure 4:**
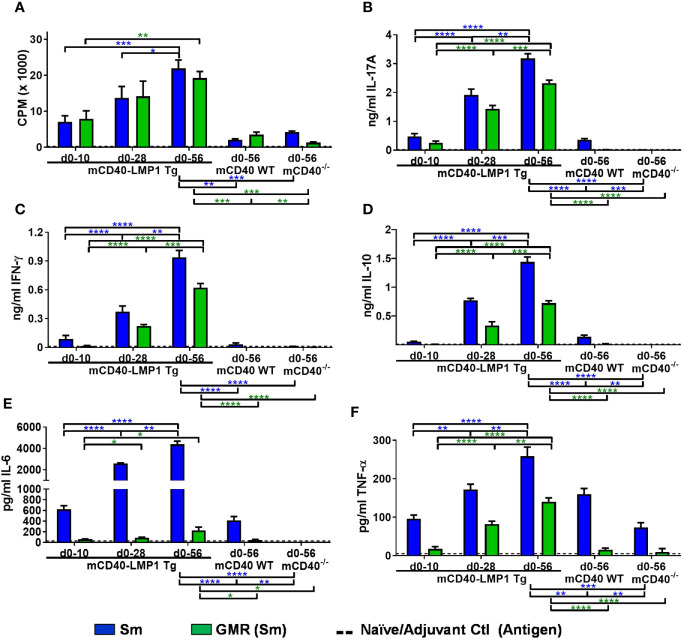
EBNA-1 immunization leads to lupus autoantigen Sm and homologous region PPPGMRPP cellular T cell responses in CD40-LMP1-Tg mice. Lymph node cells (4e6/ml; carried over from **Figure 3**) were cultured in the presence of 50 µg/ml Sm vs. 50 µg/ml PPPGMRPP (GMR; Sm antigenic epitope). Cell cultures were assessed for proliferation **(A)** and cell culture supernatant IL-17A **(B)**, IFN-γ **(C)**, IL-10 **(D)**, IL-6 **(E)**, and TNF-α **(F)**. Antigen recall response from mCD40-LMP1 Tg mice was compared to that of CD40 WT mice and CD40^-/-^ mice (**Figure 2**). Data presented as mean ± SEM. *p ≤ 0.05, **p ≤ 0.01, ***p < 0.001, ****p < 0.0001 one way ANOVA with Dunnett’s multiple comparison test. Significance between experimental groups of mice designated above bar graphs (mCD40-LMP1 mice over time) and below bar graphs (mCD40-LMP1 mice vs. CD40 WT vs. CD40^-/-^ mice); blue = Sm; green = GMR. A minimal cellular response was exhibited by naïve/adjuvant control mice (dotted line near bottom of y-axis). Antigenic stimulation vs. medium only and PMA/ionomycin is presented in **Supplementary Figure 2**.

The humoral response to EBNA-1 cross reacts to lupus autoantigens, including, Sm (35, 44, 51). Given the strong antigen-specific cellular immune response in mCD40-LMP1 mice ( (70) and **Figure 3** **/** **Supplementary Figure 1**), we asked if cellular concurrent reactivity occurred between EBNA-1 and Sm in the context of LMP1 (**Figure 4** and **Supplementary Figure 2**). We therefore measured antigen recall responses to Sm and its critical humoral epitope homologous to EBNA-1, PPPGMRPP, in the same mice where EBNA-1 antigenic recall responses were measured in **Figure 3**. Similar to the EBNA-1 response, mCD40-LMP1 mice had an enhanced cellular immune response to Sm and PPPGMRPP compared to mCD40 WT and CD40-deficient mice, with respect to both proliferation (**Figure 4A**) and cytokine secretion (**Figures 4B–F**). In addition to proliferation, Sm and PPPGMRPP antigen stimulation elicited a robust IL-17A response in mCD40-LMP1 mice immunized with EBNA-1 (**Figure 4B**). Sm and PPGMRPP also stimulated IFN-γ (**Figure 4C**) and IL-10 (**Figure 4D**) responses in EBNA-1 immunized mCD40-LMP1 mice, but to a lesser degree than the primary antigen, EBNA-1 (**Figures 3C, D**). Of note, IL-6 (**Figure 4E**) showed a response to Sm, but not PPPGMRPP, and TNF-α (**Figure 4F**) only exhibited PPPGMRPP cellular responses in mCD40-LMP1 mice. The response to Sm in mCD40 WT and CD40-deficient mice immunized with EBNA-1 suggests that these mice do mount a response to EBNA-1, and that concurrent/cross-reactivity of this response may have a CD40-independent component.

### Primary and Concurrently Reactive Response After Sm Immunization in mCD40-LMP1 vs. mCD40 WT and mCD40-Deficient Mice

**Figure 5:**
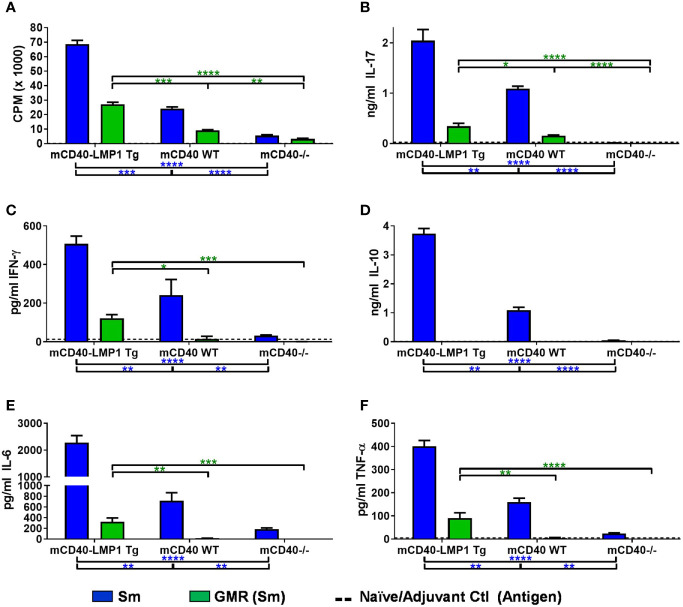
Enhanced antigen recall response to lupus autoantigen Sm and unique reactivity to homologous region PPPGMRPP in mCD40-LMP1 Tg mice. Lymph node cells (4e6/ml) were cultured in the presence of culture medium alone vs. 50 µg/ml Sm, 50 µg/ml PPPGMRPP (GMR; Sm antigenic epitope), and 5 ng/ml PMA/500ng/ml ionomycin. Cell cultures were assessed for proliferation **(A)** and cell culture supernatant IL-17A **(B)**, IFN-γ **(C)**, IL-10 **(D)**, IL-6 **(E)**, and TNF-α **(F)**. Antigen recall response from mCD40-LMP1 Tg mice was compared to that of CD40 WT mice and CD40^-/-^ mice (**Figure 2**). Data presented as mean ± SEM. *p ≤ 0.05, **p ≤ 0.01, ***p < 0.001, ****p < 0.0001 one way ANOVA with Dunnett’s multiple comparison test. Significance between experimental groups of mice designated above bar graphs (mCD40-LMP1 mice over time) and below bar graphs (mCD40-LMP1 mice vs. CD40 WT vs. CD40^-/-^ mice); blue = Sm; green = GMR. A minimal cellular response was exhibited by naïve/adjuvant control mice (dotted line near bottom of y-axis). Antigenic stimulation vs. medium only and PMA/ionomycin is presented in **Supplementary Figure 3**.

**Figure 6:**
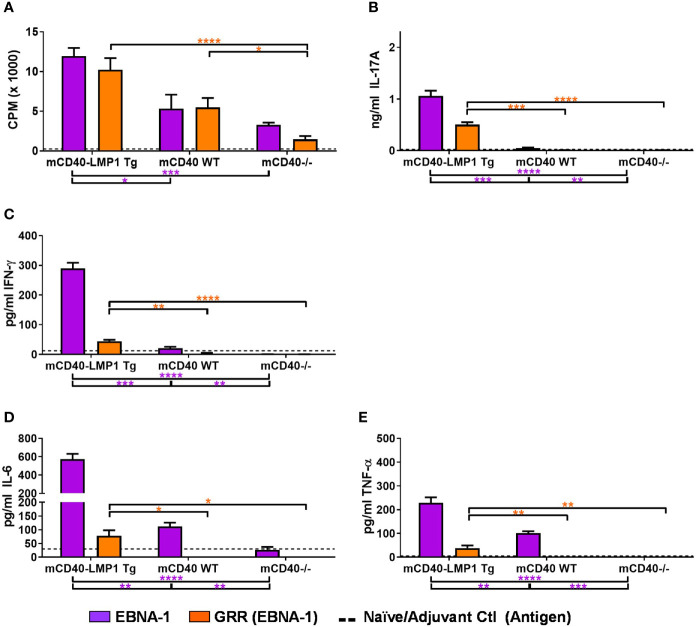
Selective Sm cross-reactivity to EBNA-1 and homologous region PPPGRRP in mCD40-LMP1 Tg mice. Lymph node cells (4e6/ml; carried over from **Figure 5**) were cultured in the presence of 50 µg/ml EBNA-1 (mosaic) vs.50 µg/ml PPPGRRP (GRR; EBNA-1 antigenic epitope). Cell cultures were assessed for proliferation **(A)** and cell culture supernatant IL-17A **(B)**, IFN-γ **(C)**, IL-6 **(D)**, and TNF-α **(E)**. Antigen recall response from mCD40-LMP1 Tg mice was compared to that of CD40 WT mice and CD40^-/-^ mice (**Figure 2**). Data presented as mean ± SEM. *p ≤ 0.05, **p ≤ 0.01, ***p < 0.001, ****p < 0.0001 one way ANOVA with Dunnett’s multiple comparison test. Significance between experimental groups of mice designated above bar graphs (mCD40-LMP1 mice over time) and below bar graphs (mCD40-LMP1 mice vs. CD40 WT vs. CD40^-/-^ mice); purple = EBNA-1; orange = GRR. A minimal cellular response was exhibited by naïve/adjuvant control mice (dotted line near bottom of y-axis). Antigenic stimulation vs. medium only and PMA/ionomycin is presented in **Supplementary Figure 4**.

Because EBNA-1 immunization of mCD40-LMP1 mice produced a strong EBNA-1 cellular immune response that concurrently reacted with Sm and its homologous epitope PPPGMRPP (**Figures 3**, **4**), we tested whether Sm immunization of mCD40-LMP1 mice would produce heightened primary (Sm) and concurrently-reactive (EBNA-1) cellular immune responses (**Figures 5**, **6** and **Supplementary Figures 3**, **4**). Indeed, compared to control mice, mCD40-LMP1 mice exhibited enhanced proliferative (**Figure 5A**) and cytokine (**Figures 5B–F**) responses to Sm as the primary antigen, as well against as its antigenic peptide PPPGMRPP, except for a lack of IL-10 after PPPGMRPP stimulation (**Figure 5D**). Unlike EBNA-1 immunization, Sm immunization did lead to a detectable Sm-specific cellular response in mCD40 WT, and to a lesser extent, mCD40-deficient mice.

Compared to the robust concurrently-reactive Sm response after EBNA-1 immunization (**Figures 3**, **4**), Sm immunization produced a more muted concurrently reactive EBNA-1 response (**Figures 5**, **6**). Nonetheless, mCD40-LMP1 Tg mice did mount a concurrently reactive proliferative and cytokine response to EBNA-1, and to a lesser extent, PPPGRRP, particularly through IL-17 (**Figure 6B**), IFN-γ (**Figure 6C**), and TNF-α (**Figure 6E**). No IL-10 was produced in any Sm-immunized mouse strain in response to EBNA-1 or PPPGRRP (**Supplementary Figure 4D**). The limited concurrently-reactive EBNA-1 cytokine response in Sm-immunized CD40 WT mice was primarily reflected by readily detectable TNF-α, while mCD40-deficient mice showed no concurrently reactive cytokine response (**Figure 6E**).

The enhanced, antigen-specific cellular response exhibited in mCD40-LMP1 mice was reflected in an increased presence of activated CD4 T cells before and after EBNA-1 immunization. Further, these mCD40-LMP1 derived T cells were more readily activated by CD3 ± CD28 (**Supplementary Figure 5**). Although follicular and marginal zone B cells are not different between mCD40-LMP1 and mCD40 WT or CD40-deficient mice, mCD40-LMP1 mice showed enrichment of a CD23lo, CD21/CD35lo immature/activated B cell population, as well as increased proliferative and cytokine responses driven by BCR ± CD154 (CD40L) stimulation (**Supplementary Figure 6**).

### Enhanced Primary and Concurrently Reactive Humoral Immune Responses Between EBNA-1 and Sm in mCD40-LMP1 Tg Mice

**Figure 7:**
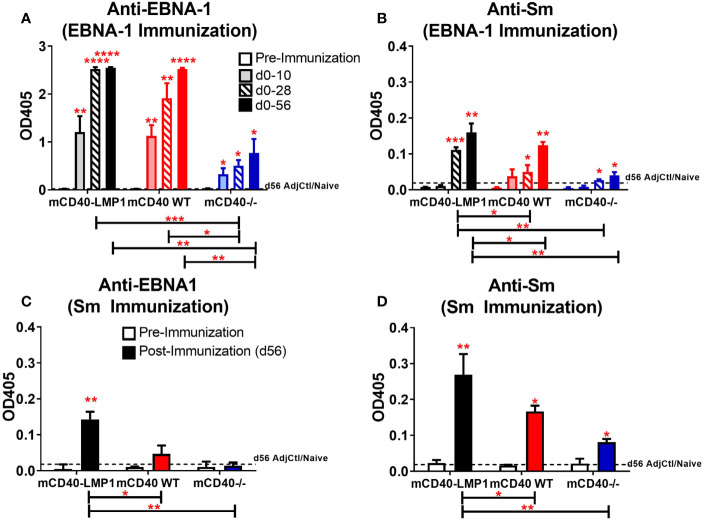
Accelerated humoral reactivity to EBNA-1 and enhanced reactivity/cross-reactivity between EBNA-1 and Sm in mCD40-LMP1 Tg mice. Sera collected at days 10, 28, and 56 **(A, B)** or day 56 only **(C, D)** post-immunization with either EBNA-1 **(A, B)** or Sm **(C, D)** were assessed for EBNA-1 (**A** and **C**, 1:1,000 serum dilution) and Sm (**B** and **D**, 1:100 serum dilution). Antibody response from mCD40-LMP1 Tg mice was compared to that of CD40 WT mice and CD40^-/-^ mice (**Figure 2**). Data presented as mean ± SEM. *p ≤ 0.05, **p ≤ 0.01, ***p < 0.001, ****p < 0.0001 one way ANOVA with Dunnett’s multiple comparison test.

Both primary and concurrent, cross-reactive antibody specificities to EBNA-1 and Sm have been observed in SLE (19, 35, 39) and EBNA-1 drives a strong humoral immune response in animal models (42, 44, 86). Therefore, we evaluated whether the anti-EBNA-1 and anti-Sm antibody responses would be enhanced in mCD40-LMP1 mice compared to mCD40 WT or CD40-deficient mice, after EBNA-1 or Sm immunization (**Figure 7**). Unlike cellular immune responses to either EBNA-1 or Sm, all strains of mice exhibited a readily detectable humoral immune response, although mCD40-deficient mice mounted significantly weaker responses, as expected. Both mCD40-LMP1 Tg and mCD40 WT mice showed a similarly strong, global anti-EBNA-1 antibody response, even as early as 10 days after initial EBNA-1 immunization (**Figure 7A**). However, mCD40-LMP1 mice mounted an earlier and more robust concurrently-reactive Sm antibody response after EBNA-1 immunization (**Figure 7B**). After Sm immunization, mCD40-LMP1 mice once again mounted a concurrently-reactive EBNA-1 response (**Figure 7C**), as well as an enhanced primary response (Sm antigen, **Figure 7D**) compared to control mice, suggesting that the dysregulated cellular immune response driven by the cytoplasmic tail of LMP1 also extends to humoral immunity.

**Figure 8:**
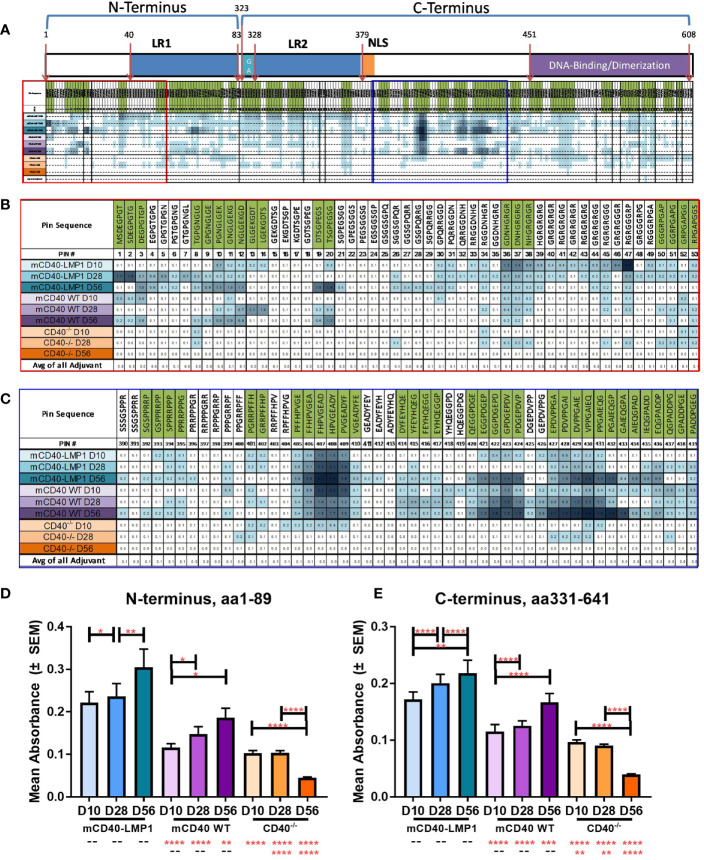
Enhanced EBNA-1 domain-specific humoral response in mCD40-LMP1 Tg mice. EBNA-1 epitope-specific humoral immunity was compared in sera from mCD40-LMP1 Tg mice vs. CD40 WT and CD40-/- mice at 10, 28, and 56 days post-immunization with EBNA-1 (vs. adjuvant/naïve control). EBNA-1 domain map and average reactivity to each EBNA-1 epitope is presented in **(A)**, with green indicating positive epitopes (≥4 SD above adjuvant control). N-terminus area of reactivity from **(A)** is presented in **(B)**. C-terminus area of reactivity from **(A)** is presented in **(C)**. Color intensity of each sample block increases with anti-EBNA-1 epitope reactivity (green shaded epitopes are considered positive if ≥4 SD above adjuvant/naïve control). Corresponding epitope mapping histograms are presented in **Supplementary Figure 7**. Mean ± SEM response to N-terminus (aa1-89, **D**) and C-terminus (aa331-641, **E**) are presented. *p ≤ 0.05, **p ≤ 0.01, ***p < 0.001, ****p < 0.0001 one way ANOVA with Dunnett’s multiple comparison test.

To further characterize the global antibody response to EBNA-1 after EBNA-1 immunization, we mapped the epitope specificity of these responses. Serum reactivity to overlapping octapeptide EBNA-1 epitopes across the EBNA-1 antigen was measured for mCD40-LMP1 Tg, mCD40WT, and mCD40-deficient mice at 10, 28, and 56 days after initial EBNA-1 immunization, compared to adjuvant controls (**Figure 8** and **Supplementary Figure 7**). The patterns of serum interactions across EBNA-1 antigen domains (**Figure 8A**) showed particular regions of reactivity within the N-terminus (one region displayed in **Figure 8B**) and C-terminus (one region displayed in **Figure 8C**) for mCD40-LMP1 Tg mice, mCD40 WT mice, or both. Both mCD40-LMP1 Tg and mCD40 WT mice showed increased responses across the N-terminus (**Figure 8D**) and C-terminus (**Figure 8E**) over time (with additional time/EBNA-1 booster immunizations); mCD40-LMP1 mice displayed an enhanced immune response across all time points. Conversely, CD40-deficient mice had a decreasing response over time after initial EBNA-1 immunization, suggesting that an initial CD40-independent antibody response converted to a primarily CD40-dependent response over time.

**Figure 9:**
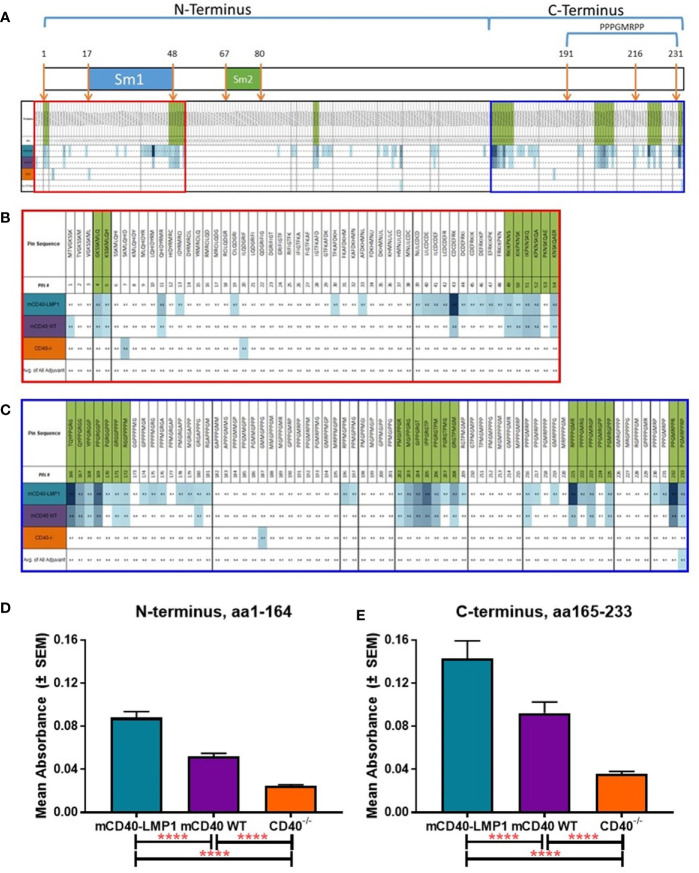
Enhanced Sm BB’ domain-specific humoral response in mCD40-LMP1 Tg mice. Sm BB’ epitope-specific humoral immunity was compared in sera from mCD40-LMP1 Tg mice vs. CD40 WT and CD40-/- mice at 56 days post-immunization with Sm antigen (vs. adjuvant/naïve control). Sm BB’ domain map and average reactivity to each Sm BB’ epitope is presented in **(A)**, with green indicating positive epitopes (≥4 SD above adjuvant control). N-terminus area of reactivity from **(A)** is presented in **(B)**. C-terminus area of reactivity from **(A)** is presented in **(C)**. Color intensity of each sample block increases with anti-Sm BB’ epitope reactivity (green shaded epitopes are considered positive if ≥4 SD above adjuvant/naïve control). Corresponding epitope mapping histograms are presented in **Supplementary Figure 8**. Mean ± SEM response to N-terminus (aa1-164, **D**) and C-terminus (aa165-233, **E**) are presented. ****p < 0.0001 one way ANOVA with Dunnett’s multiple comparison test.

Similar serum reactivity to overlapping octapeptide epitopes within the Sm BB’ antigen was measured for mCD40-LMP1 Tg, mCD40WT, and mCD40-deficient mice 56 days after initial Sm immunization, compared to adjuvant controls (**Figure 9** and **Supplementary Figure 8**). Regions of reactivity to Sm BB’ domains (**Figure 9A**) showed enhanced reactivity in Sm-immunized mice across the Sm1 region in the N-terminus (**Figure 9B**), with additional reactivity across the C-terminus, including in the PPPGMRPP antigenic region (**Figure 9C**). Similar to EBNA-1 immunization, immunizing with Sm led to a significantly increased humoral response across both the N-terminus (**Figure 9D**) and the C-terminus (**Figure 9E**) in mCD40-LMP1 Tg mice compared to mCD40 WT and CD40-deficient mice; mCD40 WT mice also mounted a significantly greater anti-Sm response across both N- and C-terminal regions compared to CD40-deficient mice. That CD40-deficient mice mounted a small, but measurable response suggests a CD40-independent component to the anti-Sm humoral immune response.

### Autoimmune Phenotype in mCD40-LMP1 Tg vs. mCD40 WT Mice in Response to EBNA-1 Immunization

**Figure 10:**
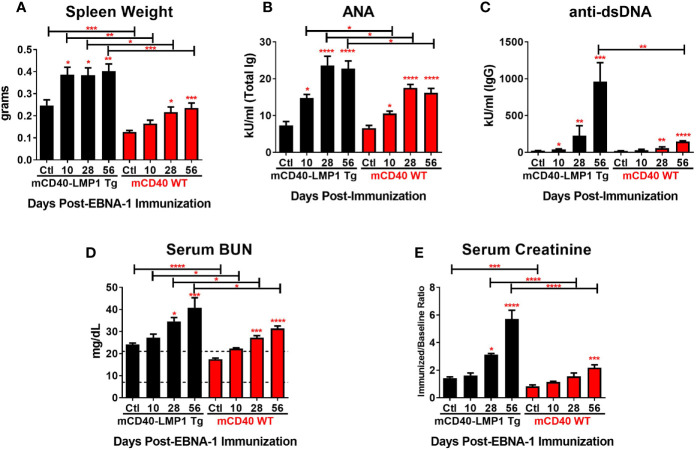
Increased spleen size, autoantibodies, and renal dysfunction in mCD40-LMP1 vs. mCD40 WT over time after EBNA-1 immunization. Mean ± SEM spleen size **(A)**, serum ANA **(B)**, serum anti-dsDNA **(C)**, serum BUN **(D)**, and serum creatinine **(E)** levels at 10, 28, and 56 days post-EBNA1 immunization (vs. adjuvant/naïve control) in mCD40-LMP1 Tg vs. mCD40 WT mice. *p ≤ 0.05, **p ≤ 0.01, ***p < 0.001, ****p < 0.0001 one way ANOVA with Dunnett’s multiple comparison test.

Unimmunized mCD40-LMP1 Tg mice show a mild autoimmune phenotype (71) that can be pushed to an inflammatory arthritis phenotype in the context of specific antigen, type II collagen (70), while the congenic mCD40 WT and mCD40-deficient strains are not prone to lupus-like disease. Given the enhanced cellular responses, humoral immunity, and dual-reactivity to lupus autoantigen Sm in mCD40-LMP1 Tg mice immunized with EBNA-1, we assessed the presence of other lupus-like features in this mouse strain (**Figure 10**). As expected, adjuvant/naïve mCD40-LMP1 Tg mice had enlarged spleens compared to mCD40 WT mice (**Figure 10A**). EBNA-1 immunization resulted in increased spleen weight in both strains of mice, but to a greater extent in the mCD40-LMP1 Tg mice. With respect to autoantibodies, both mCD40-LMP1 Tg and mCD40 WT mice exhibited increased ANA levels after EBNA-1 immunization, and this was enhanced in the mCD40-LMP1 Tg mice (**Figure 10B**). In particular, anti-dsDNA autoantibodies were markedly elevated in mCD40-LMP1 Tg mice 56 days after initial EBNA-1 immunization (**Figure 10C**). Given that anti-dsDNA is associated with lupus nephritis in SLE patients and select lupus nephritis-like mouse models (10), we also assessed serum BUN (**Figure 10D**) and creatinine (**Figure 10E**) levels. Both mCD40-LMP1 Tg and mCD40 WT mice showed increases in BUN (**Figure 10D**) and creatinine (**Figure 10E**) over time, particularly 56 days after initial EBNA-1 immunization in mCD40-LMP1 mice. However, no overt renal pathology nor areas of inflammatory cell recruitment were observed upon histological examination in either strain (data not shown). These data suggest that while some aspects of lupus-associated autoimmunity seen in mCD40-LMP1 Tg mice, enhanced with EBNA-1 immunization, that this may not be enough to drive classic immune complex glomerulonephritis in the time period assessed.

## Discussion

More complete understanding of immune dysregulation in SLE will facilitate proactive interventions with the potential to delay and minimize transition to disease classification, clinical disease flare, and permanent organ damage (5, 92). Despite the ability of a multitude of studies to elucidate genetic risk and highlight immune parameters that may be influenced by genetic variance (12, 13), genetic variation alone incompletely explains lupus pathogenesis. Turning our attention to environmental factors such as EBV with its latent immune mimics has the potential to help us further identify underlying mechanisms of immune dysregulation and opportunities for intervention. The current study expounds on the ability of the EBV-encoded functional immune mimic, LMP1, and molecular mimic, EBNA-1, to dysregulate both cellular and humoral immunity, resulting in reactivity to the SLE-associated autoantigen Sm.

Preclinical SLE is marked by the development of cross-reactive antibodies recognizing both EBNA-1 and autoantigens. Similarly, when immunized with EBNA-1 or Sm, B6 mice expressing WT mCD40 mount both a primary humoral response against the immunizing antigen and a cross-reactive response to Sm or EBNA-1, respectively. In mCD40-LMP1 Tg mice, where the cytoplasmic tail of EBV-encoded CD40 mimic LMP1 drives dysregulated signaling, this response is enhanced, particularly to Sm (primary) and EBNA-1→Sm and Sm→ EBNA-1 concurrent reactivity. Although mCD40 and mCD40-LMP1 mice exhibited a similar response to total EBNA-1, particularly in the C-terminus near the homologous epitope PPPGRRP (aa398-404), mCD40-LMP1 Tg mice had increased reactivity across the N- and C-terminal domains. This was also the case in mCD40-LMP1 Tg mice immunized directly with Sm, with increased reactivity across both N- and C-terminal domains of Sm BB’, particularly in the C-terminus near its homologous epitope PPPGMRPP (aa 191-231). Increased epitope reactivity may allow for enhanced epitope spreading and molecular mimicry/cross-reactivity to lupus associated autoantigens such as Sm. Of note, the modest antibody response to EBNA-1 and Sm in CD40-deficient mice suggests that part of the humoral immune response to these antigens was CD40-independent. The areas of EBNA-1 reactivity were far smaller, but overlapping with mCD40 WT or mCD40-LMP1. A T-independent component of humoral immunity to T-dependent antigens has been demonstrated and likely relies on another TNF-R superfamily member, BLyS/BAFF (93–95).

This study used an immunization protocol designed to induce EBNA-1 humoral immunity in animal models (86), similar to what is observed in SLE patients (19, 39). In addition to an enhanced humoral response, using this immunization strategy in mCD40-LMP1 Tg mice resulted in an enhanced cellular response to EBNA-1/PPPGRRP and Sm/PPPGMRPP, particularly with respect to proliferation, IFN-γ (Th1), and especially IL-17 (Th17) responses, as well as IL-6 and TNF-α. This is not unlike what was observed in the context of type II collagen immunization in the collagen-induced inflammatory arthritis model (70). Of note, the IL-10 response lagged behind other mediators assessed, had less reactivity to the EBNA-1 homologous epitope PPPGRRP, and had no reactivity to the Sm homologous epitope PPPGMRPP. It is possible that the regulatory IL-10 response occurs later than the pro-inflammatory mediator response or that the reactive antigenic region(s) driving an IL-10 response lie(s) outside of the homologous reactive domain for EBNA-1 and Sm. A similar lack of reactivity to the EBNA-1 and Sm homologous domains was also observed with IL-6 secretion. Given that naïve mCD40-LMP1 Tg mice already have increased systemic levels of IL-6 (71), the peptide antigen signal may not be sufficient to drive additional IL-6 production, yet allows for downstream IL-17A secretion. Alternatively, like IL-10, the antigenic region that drives IL-6 production may be outside of the EBNA-1 and Sm homologous domains.

The ability of LMP1 to drive a cellular, concurrently reactive response between EBNA-1 and Sm *in vivo* suggests a possible route for EBV to contribute to cellular molecular mimicry and immune pathway dysregulation. In the present study, EBNA-1 to Sm dual-reactivity was more robust than Sm to EBNA-1 dual-reactivity, suggesting that EBNA-1 drives concurrently reactive cellular immunity to Sm, and not the reverse. It is not unusual for concurrent, cross-reactive T-lymphocytes to drive immune and autoimmune processes (96). A cellular immune response to EBNA-1 that cross reacts with autoantigen (myelin) has been demonstrated in multiple sclerosis (97–100), despite the common lack of T-lymphocyte control of EBV infection in multiple sclerosis (101, 102) and SLE (103, 104). A cross-reactive cellular immune response to EBNA-1 has yet to be demonstrated in human SLE, but may be best detected during preclinical SLE when cellular immune dysregulation first gives rise to humoral autoimmunity (2–4, 17). Additionally, cross-reactive cellular immunity with EBNA-1 may be apparent during periods of EBV reactivation, when PBMCs are likely to express EBNA-1 and LMP1 (17), especially since LMP1 positive PBMCs coincide directly with immune dysregulation that leads to clinical disease flare (34, 105). Together, our current and previous findings suggest that LMP1 contributes to immune dysregulation that may set the stage for SLE pathogenesis. Sustained and dysregulated cellular immunity driven by LMP1 may allow for a break in tolerance that allows for the production of SLE-associated autoantibodies, including ANA and anti-dsDNA (62, 67, 69, 71). The concurrent expression of EBNA-1 and its role as a molecular mimic may then contribute to accumulation of additional SLE-associated autoantibody specificities, including Sm (9, 19, 35, 39).

Although mCD40-LMP1 Tg mice immunized with EBNA-1 developed ANA and anti-dsDNA autoantibodies, as well as some renal dysfunction with increased BUN and creatinine, no overt renal pathology was noted on histological examination. It is possible that the mCD40-LMP1 Tg mice were just starting to develop nephritis 56 days after initial EBNA-1 immunization and may have developed overt renal pathology if given more time. Alternatively, the B6 strain may be resistant to immune complex glomerulonephritis, thus requiring additional genetic influence even in the context of mCD40-LMP1. Phenotypically, naïve mCD40-LMP1 Tg mice appear similar to B6.Sle2 mice, which exhibit polyclonal antibodies and activated T-cell immunity, but require genes from B6.Sle1 mice to develop overt nephritis (106–108). Crossing mCD40-LMP1 Tg mice with B6.Sle1 mice, but not B6.Sle3 mice, accelerates autoimmunity, including increased cellular immunity, development of anti-dsDNA autoantibodies, and overt renal pathology evidenced by glomerular inflammatory infiltrates (69). Further, LMP1 is expressed in the kidneys of human SLE patients, particularly patients who are positive for anti-Sm autoantibodies (37, 109). This suggests the possibility that enhanced and dysregulated cellular immunity associated with LMP1 functional mimicry (62, 67, 69, 71) may foster anti-Sm and anti-dsDNA autoantibody specificities associated with EBNA-1 molecular mimicry (35, 42–44) to propel some aspects of immune complex-driven lupus nephritis.

**Figure 11:**
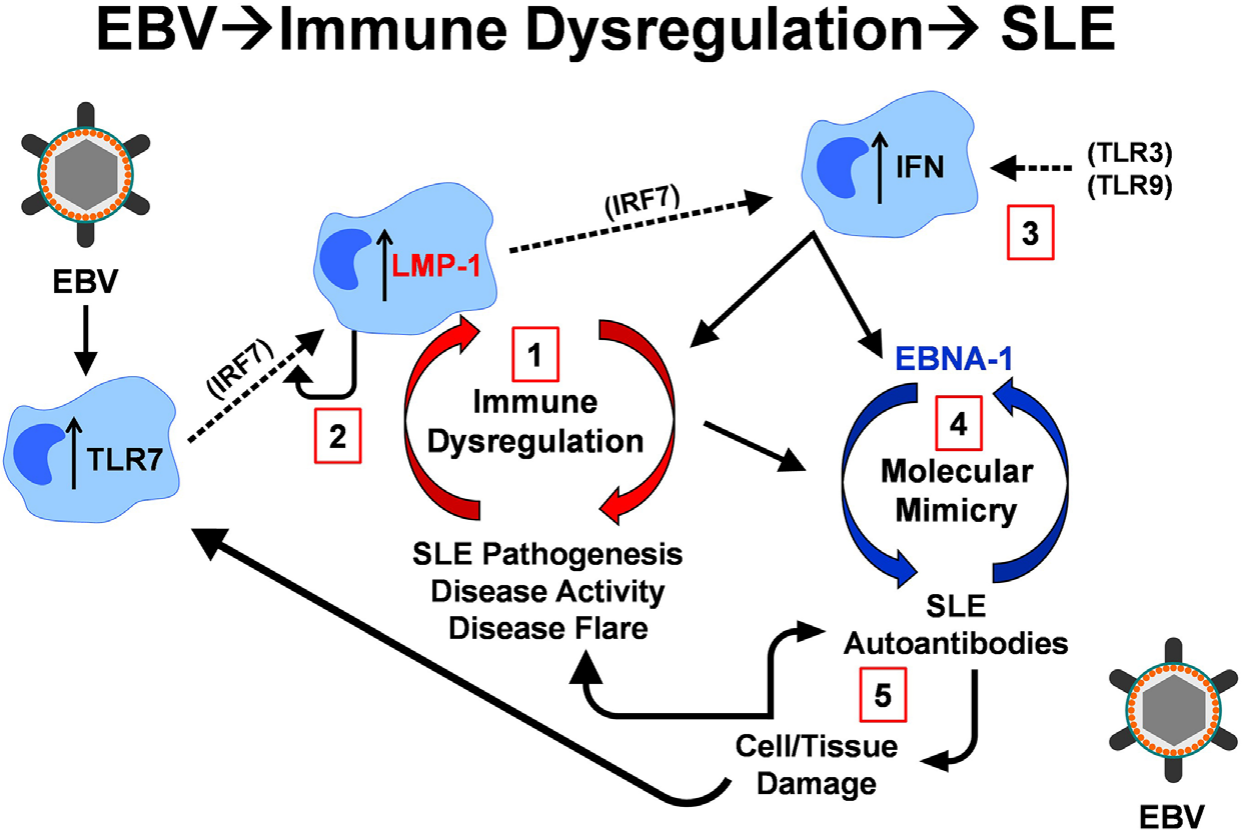
Feed forward model of LMP1 expression, immune dysregulation, and SLE autoimmunity. EBV infection can drive increased TLR7 expression *via* IRF7, which also stimulates increased LMP1 expression. LMP1, in turn, stimulates IRF7 *via* RIP to continue this positive autoregulatory loop **1**. The very immune dysregulation (innate and adaptive) that LMP1 propels also upregulates LMP1 expression **2**. In addition, LMP1 drives interferon (IFN) production *via* IRF7, in conjunction with other TLRs, including TLR3 and TLR9 **3**. Type I IFNs (innate response), in addition to the adaptive immune response that LMP1 promotes, can contribute to T cell-mediated antibody/autoantibody production, allowing for the cross-reactive, molecular mimic response between EBNA-1 and lupus autoantigens Ro, Sm, and RNP **4**. Autoantibodies, in conjunction with ongoing inflammation, lead to cell and tissue damage, releasing additional lupus-associated autoantigens that interact with TLR7 and continue to drive cellular and humoral autoimmunity **5**.

We propose that LMP1, in potential conjunction with genetic risk (17, 34, 69, 105), may contribute to immune dysregulation that fosters broken tolerance, enhancing EBNA-1 molecular mimicry and fueling autoantibody production, downstream cellular and tissue damage, and SLE pathogenesis. Although findings in the current study were driven by an mCD40-LMP1 hybrid molecule in the absence of CD40, similar cellular and humoral immune dysregulation has been noted in both *in vitro* (65) and *in vivo* (69) mouse studies in the presence of endogenous CD40, as well as in human patients with confirmed LMP1 expression (34, 110–114). Together, these findings suggest a model whereby EBV-encoded latent immune mimics initiate a network of feed-forward loops that contribute to SLE pathogenesis with LMP1 driven immune dysregulation, and EBNA-1 stimulated autoantibody production (**Figure 11**).

A positive autoregulatory loop that maintains LMP1 expression (**Figure 11**-1) could perpetuate this cycle. Both SLE-associated genetic polymorphisms (12, 13) and EBV infection can upregulate TLR7 expression *via* IRF7 (115). TLR7 stimulates LMP1 expression (112), and LMP1, in turn, stimulates IRF7 *via* RIP, promoting further LMP1 expression. The dysregulation of immune mediators by LMP1 further promotes LMP1 expression (34, 110–112, 116) (**Figure 11**-2). Of particular interest, the regulatory mediator IL-10, which is upregulated during periods of non-flare in SLE patients (6, 7), promotes LMP1 expression (110), which then has the potential to drive inflammatory immune dysregulation leading to a subsequent period of increased clinical disease activity and flare (34, 105).

LMP1 drives additional forms of immune dysregulation that contribute to SLE disease pathogenesis (3), clinical disease activity and flare (5–7), including type I IFN and Th1-, Th2-, and Th17-type immunity. Type I IFN is produced in response to LMP1-mediated IRF7 stimulation (84), in conjunction with other TLRs, including TLR3 and TLR9 (117–120) (**Figure 11**-3). Together, type I IFNs (innate response) and the adaptive immune responses enhanced by LMP1 [current study and (70, 113, 114, 116)] can contribute to T cell-mediated antibody/autoantibody production, facilitating cross-reactive responses between molecular mimic EBNA-1 and lupus autoantigens (35) (**Figure 11**-4). In addition to our findings in the current study, we and others have demonstrated cross-reactivity between EBNA-1 and lupus autoantigens, both in animal models and human SLE patients, including Ro/SSA (19, 39, 40, 86), Sm (19, 39, 44, 121), RNP (39, 51, 86), and dsDNA (39, 40, 42–44). Autoantibodies and ongoing inflammation (122) cause cellular and tissue damage that releases more lupus associated autoantigens, which can interact with TLR7 to further propagate cellular and humoral autoimmunity (112, 117, 123–126) (**Figure 11**-5).

In addition to cellular expression of LMP1, the LMP1 transmembrane domain enables extracellular expression on vesicles and exosomes (127), where it can be internalized (128), including by dendritic cells (129). This allows for LMP1-induced cellular proliferation and activation (128, 130, 131), as well as antibody production and class-switching in non-infected B cells (132). This would allow for LMP1 expression in cells other than B-lymphocytes [and epithelial cells, which are also tropic for EBV (133)] and drive additional pathogenicity.

Our findings suggest that LMP1 can both promote cellular immune dysregulation and potentiate EBNA-1 humoral immunity and dual-reactivity with the lupus autoantigen Sm. Such dysregulation may be necessary, yet insufficient, to explain SLE pathogenesis. Over 90% of the general population is EBV seropositive (134), yet only a subset of individuals develop SLE or other autoimmune diseases. Indeed, EBNA-1 molecular mimicry and LMP1-mediated immune dysregulation have been noted in patients with mononucleosis (51, 135–137), but this does not lead to autoimmune disease in most patients. It is possible that immune dysregulation fostered by EBV latent mimics provides a break in immune tolerance that creates an opportunity for SLE-associated genetic risk variants to drive SLE pathogenesis (12, 30, 138–140). In a mouse model of lupus-like disease that associates phenotype with genetic risk [B6.Sle1.Sle2.Sle3 mice (107)], we have previously demonstrated that mCD40-LMP1 Tg mice accelerate lupus-like autoimmunity in B6.Sle1, but not B6.Sle3 mice [mCD40-LMP1 Tg mice are phenotypically similar to B6.Sle2 mice (71, 107)], including histologic evidence of glomerulonephritis) (69). Further, SLE patients experiencing heightened clinical disease activity and flare have been shown to exhibit an altered type I IFN gene signature that is associated with LMP1 expression in PBMCs (34). Immune dysregulation that contributes to SLE pathogenesis, clinical disease activity, and organ damage may be further augmented by lifestyle and other environmental factors, including smoking (141–144), UV exposure (145–147), and changes in gut microbiome (148–150). Future studies that further elucidate the relationship in gene-environment interactions, including EBV-encoded latent mimics, have the potential to better define windows of therapeutic opportunity for targeted treatments.

## Data Availability Statement

The raw data supporting the conclusions of this article will be made available by the authors, without undue reservation.

## Ethics Statement

The animal study was reviewed and approved by Oklahoma Medical Research Foundation Institutional Animal Care & Use Committee (IACUC).

## Author Contributions

MM managed mouse colonies, designed and carried out experiments, completed data analysis, and principally wrote manuscript. JA and TG provided technical support. LS and GB provided transgenic mice and Hi5 (WT and mCD154) insect cells, as well as experimental and editorial guidance. JJ provided additional support, as well as experimental and editorial guidance. All authors contributed to the article and approved the submitted version.

## Funding

This work was supported by the National Institute of Arthritis and Musculoskeletal and Skin Diseases and the National Institute of General Medical Sciences through the NIH (P30RR031152, UM1AI144292, P30AR073750, and U54GM104938). The contents are solely the responsibility of the authors and do not necessarily represent the official views of the NIH or one of its institutes. This work was also supported by the OMRF Lou C. Kerr Chair in Biomedical Research to JJ.

## Conflict of Interest

The authors declare that the research was conducted in the absence of any commercial or financial relationships that could be construed as a potential conflict of interest.
