# Supplementary material for: Epstein-Barr Functional Mimicry: Pathogenicity of Oncogenic Latent Membrane Protein-1 in Systemic Lupus Erythematosus and Autoimmunity
Source: Front Immunol. 2021 Feb 3;11:606936. doi: 10.3389/fimmu.2020.606936 (PMC7886997; doi:10.3389/fimmu.2020.606936)
Supplement: Supplementary file 1 [file DataSheet_1.docx]

Supplementary Material

**Supplementary Figure 1. Enhanced antigen recall response to EBNA-1 and homologous region PPPGRRP in mCD40-LMP1 Tg mice.** Lymph node cells (4x 10^6^/ml) were cultured in the presence of culture medium alone vs. 50 µg/ml EBNA-1 (mosaic), 50 µg/ml PPPGRRP (GRR; EBNA-1 antigenic epitope), and 5 ng/ml PMA/500ng/ml ionomycin. Cell cultures were assessed for proliferation (**A**) and cell culture supernatant IL-17A (**B**), IFN-γ (**C**), IL-10 (**D**), IL-6 (**E**), and TNF-α (**F**). Antigen recall response from mCD40-LMP1 Tg mice was compared to that of CD40 WT mice and CD40^-/-^ mice (**Figure 2**). Data presented as mean ± SEM. **p≤0.05*, ***p≤0.01*, ****p<0.001*, *****p<0.0001* one way ANOVA with Dunnett’s multiple comparison test. Signicance compared to medium only designated above bar graphs (black). Significance between experimental groups of mice designated below bar graphs (purple = EBNA-1; orange = GRR). A minimal cellular response was exhibited by naïve/adjuvant control mice (dotted line near bottom of y-axis). A minimal cellular response was exhibited by d0-10 and d0-28 mCD40 WT and CD40 deficient mice; only d0-56 data are shown.

**Supplementary Figure 2. EBNA-1 immunization leads to lupus autoantigen Sm and homologous region PPPGMRPP cellular T cell responses in CD40-LMP1-Tg mice.** Lymph node cells (4e6/ml) were cultured in the presence of culture medium alone (carried over from Figure 3) vs. 50 µg/ml Sm, 50 µg/ml PPPGMRPP (GMR; Sm antigenic epitope), and 5 ng/ml PMA/500ng/ml ionomycin (carried over from **Figure 3**). Cell cultures were assessed for proliferation (**A**) and cell culture supernatant IL-17A (**B**), IFN-γ (**C**), IL-10 (**D**), IL-6 (**E**), and TNF-α (**F**). Antigen recall response from mCD40-LMP1 Tg mice was compared to that of CD40 WT mice and CD40^-/-^ mice (**Figure 2**). Data presented as mean ± SEM. **p≤0.05*, ***p≤0.01*, ****p<0.001*, *****p<0.0001* one way ANOVA with Dunnett’s multiple comparison test. Signicance compared to medium only designated above bar graphs (black). Significance between experimental groups of mice designated below bar graphs (blue = Sm; green = GMR). A minimal cellular response was exhibited by naïve/adjuvant control mice (dotted line near bottom of y-axis).

**Supplementary Figure 3. Enhanced antigen recall response to lupus autoantigen Sm and unique reactivity to homologous region PPPGMRPP in mCD40-LMP1 Tg mice.** Lymph node cells (4e6/ml) were cultured in the presence of culture medium alone vs. 50 µg/ml Sm, 50 µg/ml PPPGMRPP (GMR; Sm antigenic epitope), and 5 ng/ml PMA/500ng/ml ionomycin. Cell cultures were assessed for proliferation (**A**) and cell culture supernatant IL-17A (**B**), IFN-γ (**C**), IL-10 (**D**), IL-6 (**E**), and TNF-α (**F**). Antigen recall response from mCD40-LMP1 Tg mice was compared to that of CD40 WT mice and CD40^-/-^ mice (**Figure 2**). Data presented as mean ± SEM. **p≤0.05*, ***p≤0.01*, ****p<0.001*, *****p<0.0001* one way ANOVA with Dunnett’s multiple comparison test. Signicance compared to medium only designated above bar graphs (black). Significance between experimental groups of mice designated below bar graphs (blue = Sm; green = GMR). A minimal cellular response was exhibited by naïve/adjuvant control mice (dotted line near bottom of y-axis).

**Supplementary Figure 4. Selective Sm cross-reactivity to EBNA-1 and homologous region PPPGRRP in mCD40-LMP1 Tg mice.** Lymph node cells (4e6/ml) were cultured in the presence of culture medium alone (carried over from **Figure 5**) vs. 50 µg/ml EBNA-1 (mosaic), 50 µg/ml PPPGRRP (GRR; EBNA-1 antigenic epitope), and 5 ng/ml PMA/500ng/ml ionomycin (carried over from **Figure 5**). Cell cultures were assessed for proliferation (**A**) and cell culture supernatant IL-17A (**B**), IFN-γ (**C**), IL-10 (**D**), IL-6 (**E**), and TNF-α (**F**). Antigen recall response from mCD40-LMP1 Tg mice was compared to that of CD40 WT mice and CD40^-/-^ mice (**Figure 2**). Data presented as mean ± SEM. **p≤0.05*, ***p≤0.01*, ****p<0.001*, *****p<0.0001* one way ANOVA with Dunnett’s multiple comparison test. Signicance compared to medium only designated above bar graphs (black). Significance between experimental groups of mice designated below bar graphs (purple = EBNA-1; orange = GRR). A minimal cellular response was exhibited by naïve/adjuvant control mice (dotted line near bottom of y-axis).

**Supplementary Figure 5.** Increase in activated CD4 T-lymphocytes in mCD40-LMP1 Tg mice, enhanced with EBNA-1 immunization.

**A**. Splenocytes were evaluated for the relative presence of naïve (CD44lo, CD62Lhi) and activated (CD44hi, CD62Llo) CD3/CD4-positive T-lymphocytes from mCD40-LMP1 Tg, mCD40 WT, and mCD40^-/-^ administered adjuvant only (or naïve, Ctl) vs. immunized/boosted with EBNA-1 as described in *Materials and Methods*. For flow cytometry staining, a total of 1x10^6^ splenocytes (single-cell suspension, erythrocytes lysed with ACK buffer, followed by multiple washes in ice-cold media) were stained with directly conjugated fluorescent Abs after blocking Fc receptors with Mouse BD Fc Block^TM^ (BD Biosciences, San Jose, CA). Stained cells were washed and fixed using BD Cytofix/Cytoperm (BD Biosciences) according to the manufacturer’s instructions. Data were collected on a FACSCalibur (Becton Dickinson, Mountain View, CA) using CellQuest software (CellQuest, Tampa, FL). The results were analyzed usingFlowJo software (Tree Star, San Carlos, CA), gating first on live cells. PerCP-conjugated anti-CD3 and isotype control antibodies were purchased from BD Biosciences. PE-conjugated anti-CD4, FITC-conjugated anti-CD44, and APC-conjugated anti-CD62L were purchased from eBioscience/ Thermo Fisher Scientific, Waltham, MA.

**B**. Proliferation of T-lymphocytes from mCD40-LMP1 Tg, mCD40 WT, and mCD40^-/-^ mice administered adjuvant only (or naïve, Ctl) vs. immunized/boosted with EBNA-1 as described in *Materials and Methods*. Splenic T-lymphocytes were enriched by negative selection using Miltenyi Pan T cell isolation kit and Quadro-MACS magnet/column system per manufacturer’s instructions (Miltenyi Biotec, Auburn, CA). Enriched T-lymphocytes (2 x 10^6^/ml) were cultured in the presence of culture medium only (as described in *Materials and Methods*) or plate-bound anti-CD3 (0.5 µg/ml; clone OKT3, eBioscience/Thermo Fisher Scientific) ± anti-CD28 (5 µg/ml, clone CD28.2, eBioscience/Thermo Fisher Scientific). Cellular proliferation was determined as described in *Materials and Methods*.

**A**-**B**. Data presented as mean ± SEM. **p≤0.05*, ***p≤0.01*, ****p<0.001*, *****p<0.0001* Student’s paired t-test (**A**. EBNA-1 vs Ctl) and one way ANOVA with Dunnett’s multiple comparison test (**A-B**, mCD40-LMP1Tg vs. mCD40 WT vs. mCD40^-/-^).

**Supplementary Figure 6.** Increase in immature/activated splenic B-lymphocytes in mCD40-LMP1 Tg mice, enhanced with EBNA-1 immunization.

**A**. Splenic B-lymphocytes (B220^+^) were evaluated for the presence of immature/naïve (Imm; CD23^lo^, CD21/35^lo^), follicular (FO; CD23^hi^, CD21/35^lo^), and marginal zone (MZ; CD23^lo^, CD21/35^hi^) populations. Representative flow cytometry gating plots for mCD40-LMP1 Tg and mCD40 WT mice are shown. Flow cytometry was performed as described in *Supplementary Figure 1.* PerCP-conjugated anti-B220 and isotype control antibodies were purchased from BD Biosciences. PE-conjugated anti-CD21 and anti-CD35 and FITC-conjugated anti-CD23 were purchased from eBioscience/ Thermo Fisher Scientific, Waltham, MA.

**B**. Immature/activated, but not follicular (FO) or marginal zone (MZ) B-lymphocytes are enriched in mCD40-LMP-1 Tg mice, with increased enrichment 56 days after initial EBNA-1 immunization. B220^+^ B-lymphocytes from mCD40-LMP1 Tg, mCD40 WT, and mCD40^-/-^ administered adjuvant only (or naïve, Ctl) were compared to immunized/boosted with EBNA-1 as described in *Materials and Methods*.

**C-E**. Splenic B-lymphocytes were negatively selected using anti-mouse CD43 conjugated MACS beads and Quadro-MACS magnet/column system per manufacturer’s instructions (Miltenyi Biotec). Enriched B-lymphocytes (2 x 10^6^/ml) were cultured in the presence of Hi5 insect cells (1:8 Hi5:B-lymphocytes, control), BCR stimulation (10 µg/ml goat anti-mouse IgM F(ab’)2 m-chain specific [Jackson ImmunoResearch Laboratories, West Grove, PA], Hi-5 insect cells expressing CD154 (1:8 Hi5:B-lymphocytes), the ligand for CD40, or BCR + Hi5-CD154. Hi5 insect cells grow at 26°C and rapidly die to form membrane fragments at 37°C and therefore do not overgrow cell cultures. Proliferation (**C**) was determined as described in *Materials and Methods*. IL-6 (**D**) and TNF-α (**E**) levels were determined by ELISA in 48h cell cultures as described in *Materials and Methods*.

**B-E**. Data presented as mean ± SEM. **p≤0.05*, ***p≤0.01*, ****p<0.001*, *****p<0.0001* Student’s paired t-test (**B**. EBNA-1 vs Ctl) and one way ANOVA with Dunnett’s multiple comparison test (**B-E**, mCD40-LMP1Tg vs. mCD40 WT vs. mCD40^-/-^).

**Supplementary Figure 7.** EBNA-1 octapeptide epitope mapping histograms, corresponding with *Figure 8*, in sera from mCD40-LMP1 Tg (**A**), mCD40 WT (**B**), and CD40 deficient (**C**) mice.

**Supplementary Figure 8.** Sm BB’ octapeptide epitope mapping histograms, corresponding with *Figure 9*, in sera from mCD40-LMP1 Tg (**A**), mCD40 WT (**B**), and CD40 deficient (**C**) mice.
